# Multidimensional beta‐diversity across local and regional scales in a Chinese subtropical forest: The role of forest structure

**DOI:** 10.1002/ece3.10607

**Published:** 2023-10-24

**Authors:** Zhiliang Yao, Xin Yang, Bin Wang, Xiaona Shao, Handong Wen, Yun Deng, Zhiming Zhang, Min Cao, Luxiang Lin

**Affiliations:** ^1^ CAS Key Laboratory of Tropical Forest Ecology Xishuangbanna Tropical Botanical Garden, Chinese Academy of Sciences Kunming China; ^2^ University of Chinese Academy of Sciences Beijing China; ^3^ School of Ecology and Environment Hainan University Haikou China; ^4^ School of Ecology and Environmental Sciences & Yunnan Key Laboratory for Plateau Mountain Ecology and Restoration of Degraded Environments Yunnan University Kunming China; ^5^ School of the Environment University of Windsor Windsor Canada; ^6^ National Forest Ecosystem Research Station at Ailaoshan Jingdong Yunnan China; ^7^ National Forest Ecosystem Research Station at Xishuangbanna Mengla Yunnan China

**Keywords:** beta‐diversity partition, LiDAR, scale dependence, species turnover, subtropical evergreen broad‐leaved forest

## Abstract

Beta‐diversity, or the spatio‐temporal variation in community composition, can be partitioned into turnover and nestedness components in a multidimensional framework. Forest structure, including comprehensive characteristics of vertical and horizontal complexity, strongly affects species composition and its spatial variation. However, the effects of forest structure on beta‐diversity patterns in multidimensional and multiple‐scale contexts are poorly understood. Here, we assessed beta‐diversity at local (a 20‐ha forest dynamics plot) and regional (a plot network composed of 19 1‐ha plots) scales in a Chinese subtropical evergreen broad‐leaved forest. We then evaluated the relative importance of forest structure, topography, and spatial structure on beta‐diversity and its turnover and nestedness components in taxonomic, functional, and phylogenetic dimensions at local and regional scales. We derived forest structural parameters from both unmanned aerial vehicle light detection and ranging (UAV LiDAR) data and plot inventory data. Turnover component dominated total beta‐diversity for all dimensions at the two scales. With the exception of some components (taxonomic and functional turnover at the local scale; functional nestedness at the regional scale), environmental factors (i.e., topography and forest structure) contributed more than pure spatial variation. Explanations of forest structure for beta‐diversity and its component patterns at the local scale were higher than those at the regional scale. The joint effects of spatial structure and forest structure influenced component patterns in all dimensions (except for functional turnover) to some extent at the local scale, while pure forest structure influenced taxonomic and phylogenetic nestedness patterns to some extent at the regional scale. Our results highlight the importance and scale dependence of forest structure in shaping multidimensional beta‐diversity and its component patterns. Clearly, further studies need to link forest structure directly to ecological processes (e.g., asymmetric light competition and disturbance dynamics) and explore its roles in biodiversity maintenance.

## Graphical abstract

Multidimensional beta‐diversity and its turnover and nestedness components were assessed at local and regional scales in a subtropical forest. Forest structure was derived from unmanned aerial vehicle light detection and ranging data and plot inventory data. Forest structure is a non‐negligible driver in shaping multidimensional beta‐diversity pattern.

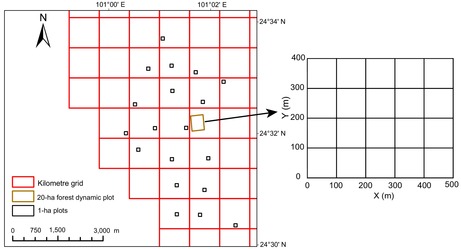

## 1 INTRODUCTION

Studying the mechanisms underlying community structure is a crucial research area in ecology. Beta‐diversity, commonly described as the variation in the identities and/or abundances of species across sample units, serves as a bridge between local diversity (i.e., alpha‐diversity) and the regional species pool (i.e., gamma‐diversity; Anderson et al., 2011; Whittaker, 1960). Previous studies examining the multiple‐scale processes that drive beta‐diversity patterns have provided considerable insights into biodiversity maintenance (Chase & Myers, 2011; Condit et al., 2002). However, ongoing debates revolve around the relative contributions of factors influencing beta‐diversity patterns and its scale effects (Myers et al., 2013; Tuomisto et al., 2003).

Dissimilarity in community composition between two sites (i.e., pairwise dissimilarity) is frequently used to measure beta‐diversity and consists of two complementary processes: species turnover component (also called species replacement) and nestedness component (Baselga, 2010, 2013; Legendre, 2014). The species turnover component involves an exchange of species identities or relative abundances among communities and may be caused by species gain or loss as a result of competition, environmental filtering, and historical factors (Legendre, 2014; Leprieur et al., 2011; Svenning et al., 2011). Conversely, the nestedness component represents the degree to which the species composition of one community is a subset of the other community and may develop from habitat filtering across environmental gradients or selective colonization (or extinction) under historical constraints (Greve et al., 2005; Ulrich et al., 2009). Although the two complementary components contribute jointly to total beta‐diversity, their relative contribution is determined by a variety of processes that affect community structure (Gianuca et al., 2017; Zhao et al., 2021). Additionally, multiple ecological factors that drive the patterns of overall beta‐diversity and its turnover and nestedness components, as well as the relative influence of these processes, vary spatially (Antão et al., 2019; Wang et al., 2018). Therefore, evaluating its opposing components and relating such patterns to multiple‐scale ecological processes, in beta‐diversity studies, offer critical insights into the mechanisms underlying observed community dissimilarities. Recently, several approaches to partitioning community dissimilarity have been proposed (Baselga, 2010, 2013; Podani & Schmera, 2011), and Baselga's additive partitioning framework has become commonly recognized by community ecologists.

Traditional beta‐diversity studies have mainly been based on differences in taxonomic composition among communities while ignoring ecological differences (e.g., evolutionary and functional differences) among species (Cadotte et al., 2019; Swenson, 2013). However, beta‐diversity dimensions that account for species functional traits and species relatedness by calculating functional and phylogenetic distance among sites may show different patterns than taxonomic beta‐diversity. For instance, the presence of many closely related species or functionally similar species among communities may lead to high species turnover in the taxonomic dimension but low turnover in the functional or phylogenetic dimension. Considering the complexities and inconsistency of beta‐diversity patterns, it is vital to integrate taxonomic, functional, and phylogenetic information about species in given communities (Gianuca et al., 2018; Mugnai et al., 2022). Moreover, Baselga's additive partitioning framework also allows the decomposition of phylogenetic and functional beta‐diversity indices into turnover and nestedness components when they are estimated by using branch lengths and convex hull volume (Leprieur et al., 2012; Villéger et al., 2013). This allows for a systematic comparison of multifaceted beta‐diversity and provides a more thorough view of community assembly and diversity maintenance determinants across ecological gradients and evolutionary histories.

Forests are undoubtedly one of the most important terrestrial ecosystems and contribute significantly to climate regulation. The vertical architecture of their constituent species is the most distinctive feature of forests. This unique feature expands niche space vertically and enables 3‐D habitat structure, providing essential habitat elements and diverse food resources for specific species (e.g., vertebrates, invertebrates, and epiphytes; Fordyce & DeVries, 2016; Nakamura et al., 2017). Additionally, 3‐D habitat structure alters microclimatic conditions, affecting fine‐scale variation and distribution of light quality and quantity, air humidity, and temperature (Onoda et al., 2014). Consequently, complex interaction effects of these microhabitat variations lead to changed resource heterogeneity and availability for trees (e.g., water, nutrients, light), potentially giving benefits to specific species that differ in their resource adaptation abilities, forming a complex forest structure.

Forest structure, including comprehensive characteristics of vertical (e.g., mean canopy height) and horizontal aspects (e.g., canopy cover), strongly affects forest biomass and productivity (Ali, 2019; Aponte et al., 2020), and multidimensional diversity (Chu et al., 2019; Thom et al., 2021; Zellweger et al., 2017). As an example, by evaluating impacts related to multiple environmental covariates, including forest structure and climate, on functional diversity, Thom et al. (2021) found that forest structure rather than climate conditions primarily determines tree species functional diversity distribution in northeastern North America. However, most studies exploring the mechanisms driving beta‐diversity patterns in the context of neutral and niche theories have primarily focused on climate, local environmental conditions, and spatial structure (e.g., Myers et al., 2013; Wang et al., 2018), but rarely considering forest structure with substantial ecological meanings. Moreover, how forest structure affects beta‐diversity patterns in multidimensional and multiscale contexts is poorly studied (but see, Nascimbene et al., 2013; Zellweger et al., 2017).

In this study, we aim to disentangle the effects of forest structure, topography, and spatial structure on multidimensional beta‐diversity (i.e., taxonomic, functional, and phylogenetic) and its turnover and nestedness components at local and regional scales in a Chinese subtropical evergreen broad‐leaved forest. Our analysis intends to unravel the ecological drivers (especially forest structure) underpinning beta‐diversity patterns. In practice, we utilized datasets collected from inventory plots and UAV LiDAR technology to quantify forest structure across two scales. Specifically, at each dimension across two scales, we examined (a) the relative contribution of turnover and nestedness components to total beta‐diversity patterns; (b) the power of forest structure to explain beta‐diversity and its component patterns; and (c) the relative importance of forest structure, topography, and spatial structure in determining patterns of beta‐diversity and its components.

## 2 MATERIALS AND METHODS

### 2.1 Study area

We carried out this study in the Xujiaba area located in the Ailaoshan National Nature Reserve, Yunnan, southwest China. As recorded by a weather station, in this area, the average annual temperature is 11.3°C and the average annual precipitation is 1778 mm. Here, at local and regional scales, we established two subtropical forest biodiversity observation platforms.

**FIGURE 1:**
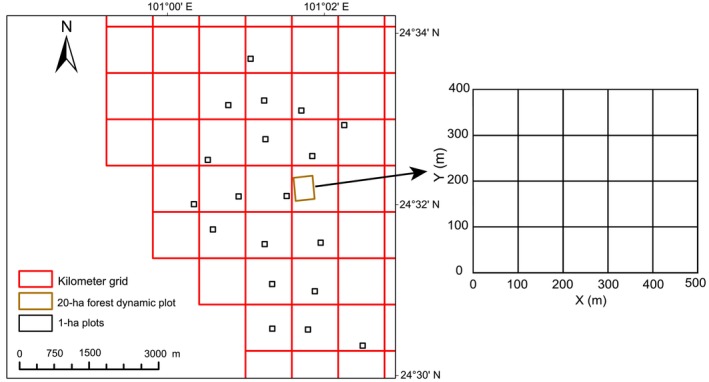
Map of the study area showing plot locations at local and regional scales in north Ailaoshan, China. At the regional scale, we established 19 1‐ha plots, forming a 1‐ha plot network. For meaningful comparisons with the 1‐ha plot network, we divided the 20‐ha forest dynamics plot at the local scale into 20, 100 × 100 m subplots.

At the local scale, following the Centre for Tropical Forest Science measurement protocols (Condit, 1998), the Ailaoshan 20‐ha forest dynamics plot (FDP) was established in 2014. Specifically, all free‐standing woody plant stems ≥1 cm DBH (diameter at breast height) were mapped, tagged, identified to species, and measured. The geographic position of the plot origin is 24°32′20″ N, 101°01′35″ E, and it is a 500 × 400 m quadrat (Figure 1). The plot location is rugged, as elevation varies from 2472 to 2628 m above sea level, and it has three ravines and three ridges.

At the regional scale, 19 1‐ha forest dynamics plots were established in northern Ailaoshan, forming a 1‐ha plot network (Figure 1). All trees ≥10 cm DBH were mapped, tagged, identified to species, and measured across all plots.

At both scales, the vegetation type across all plots is classified as subtropical mid‐mountain moist evergreen broad‐leaved forests. Thus, both have the same floral composition and are dominated by subtropical species, such as *Lithocarpus hancei* (Fagaceae), *Lithocarpus xylocarpus* (Fagaceae), *Schima noronhae* (Theaceae), and *Machilus bombycina* (Lauraceae). For meaningful comparisons with the 1‐ha plot network, we divided the Ailaoshan FDP into 20, 100 × 100 m subplots and selected only trees ≥10 cm DBH (Figure 1). Therefore, 73 and 81 species were considered at local and regional scales, respectively.

### 2.2 Functional traits and phylogenetic tree

We measured functional traits for each selected species across all plots in March 2021 and estimated a mean value at the species level for 11 key functional traits: leaf thickness (LT, cm), leaf area (LA, cm^2^), leaf dry matter content (LDMC, g g^−1^), specific leaf area (SLA, cm^2^ g^−1^), leaf chlorophyll content (LCC, SPAD value), leaf nitrogen content (leaf N, g kg^−1^), leaf carbon content (leaf C, g kg^−1^), leaf potassium content (leaf K, g kg^−1^), leaf phosphorus content (leaf P, g kg^−1^), tree maximum height (*MaxH*, m), and wood density (WD, g cm^−3^). These functional traits, which are commonly used in forest community functional diversity analyses, represent tree species performance linked to species interactions, resource use efficiency, nutrient cycling, and life history strategies (Benavides et al., 2019; Kraft et al., 2008; Yang et al., 2015). Here, we selected three to six individuals for each species. Three healthy, mature leaves were randomly collected from the outer canopy for each individual. The LT was measured using a vernier caliper, with measurements taken from the top, middle, and bottom of the leaf and averaged. The LCC was measured as the mean value of the readings at three positions (i.e., top, middle, and bottom) of a leaf using a SPAD‐502 chlorophyll meter (Konica Minolta). Fresh mass was measured using an analytical balance. Dry mass was measured after oven drying the samples at 70°C for 72 h. Each leaf was scanned to measure LA using the R package “LeafArea” (Katabuchi, 2015). LDMC was the dry mass of a leaf divided by its fresh mass and SLA was the LA divided by the dry mass. Leaf carbon (C) and nitrogen (N) contents were measured with a Dumas‐type combustion C‐N elemental analyzer (Vario MAX CN, Elementar Analysensysteme GmbH). Leaf phosphorus (P) and potassium (K) contents were determined with an inductively coupled plasma atomic‐emission spectrometer (iCAP6300, Thermo Fisher Scientific). Wood samples were taken from large branches using a tree borer, and wood density was measured by water displacement method. The *MaxH* was extracted from the Flora of China database (http://www.iplant.cn/frps). The raw trait data were transformed into a Euclidean distance matrix after being standardized with zero mean and unit standard deviation. Then, we applied principal coordinate analysis (PCoA) to reduce the dimensionality of the original trait Euclidean distance matrix and obtain orthogonal trait axes. Finally, the first four PCoA axes were used in the functional beta‐diversity calculations, and they explained 74.82% and 74.69% of all functional variability at local and regional scales, respectively (See in Table A1).

We constructed a community phylogeny for selected species at local and regional scales using chloroplast genome sequences. We extracted the coding sequences using Phylosuite software (Zhang et al., 2020). To avoid unreliable alignment, high‐sequence variability of *infA*, *ycf1*, and *ycf2* was excluded from phylogenetic analysis. We aligned the resulting 76 genes with MAFFT v7 software (Katoh & Standley, 2013), and some poorly aligned regions were adjusted manually in Geneious v11.0.2 software (Ripma et al., 2014) and then concatenated into a supermatrix with a final alignment of 60,236 bp. We constructed a maximum likelihood tree under the GTRGAMMA model using the RAxML v8.2.12 software (Stamatakis, 2014). The only two gymnosperm species, *Pinus armandii* and *Pinus kesiya*, were the outgroups in rooted trees with 1000 bootstrap replicates to evaluate nodal support. Penalized likelihood dating analysis was performed in TreePL software (Smith & O'Meara, 2012) using 1000 bootstrap replicates. Four calibration points were selected from published data (Lu et al., 2018). Finally, we summarized the age statistics for all nodes using the TreeAnnotator module of BEAST 2.5 software (Bouckaert et al., 2019).

### 2.3 Topography, forest structure, and spatial structure

UAV LiDAR data of the study plots were collected from October 2018 to February 2019 using a Velodyne LiDAR PUCK‐16 laser scanner. The flight height was set at 70 m above the canopy, and the flight velocity was controlled at about 3.6 m s^−1^. The obtained point cloud data of each plot were processed using the following procedure, namely denoising, filtering, and normalization. Concretely, the denoising method was designed to eliminate outlier points from the original data. In the filtering step, we extracted the ground points using the improved progressive triangulated irregular network (TIN) densification (IPTD) filtering algorithm (Zhao et al., 2016), and then produced a digital terrain model (DTM) from the classified ground points. In the normalization step, the elevation of every single point location was interpolated using extracted ground points, and then the raw point cloud height was subtracted from the interpolated point elevation at the corresponding location to remove the effect of topography on the point clouds. At last, the normalized point cloud data for each plot were produced. The denoising and filtering procedures were completed using LiDAR360 software (GreenValley International Inc.), and the normalization step was achieved using the *normalize_height* function in the “lidR” package (Roussel et al., 2020).

**FIGURE A1:**
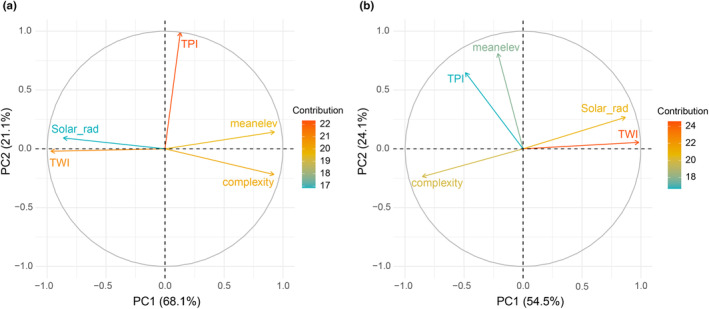
Results of PCA of five plot‐level topographic variables at local (a) and regional (b) scales. Numbers in parentheses represent the percentages of the total variation accounted by the corresponding principal component. The abbreviations of variables followed Table A2.

A DTM with 10 m resolution for each plot was interpolated by extracted ground points using the inverse distance weighted *k*‐nearest neighbor algorithm. Based on the DTM, we acquired a set of topographic variables for each plot at two scales. Specifically, topographic variables in each DTM 10 × 10 m quadrat were calculated, and then we took the mean values of the above quadrat‐level topographic variables for estimating four plot‐level topographic variables: mean elevation; topographic position index (TPI), which measures how curved the terrain is in relation to its surroundings and is either negative for concave terrain or positive for convex terrain (Jucker et al., 2018); topographic wetness index (TWI), which is expressed by the ratio of each quadrat's upslope area to its local slope and can capture important aspects of wetness (Punchi‐Manage et al., 2013); solar radiation aspect index, which quantifies the amount of solar radiation and correlates with slope. Additionally, we calculated topographic complexity (the surface‐to‐planimetric area ratio) for each plot. In total, we obtained five plot‐level topographic variables and synthesized environmental heterogeneity in terms of topography for each plot. Descriptions of these parameters are presented in Table A2. Computations were completed using the “raster” (Hijmans, 2022) and “spatialEco” (Evans & Murphy, 2021) packages. We used the orthogonal axes obtained by principal component analysis (PCA) with the correlation matrix of the topographic variables to circumvent the influence of collinearity among the topographic variables on the analysis results. Finally, we selected the first two axes, which explained 89.22% and 78.64% of all topographic variation at local and regional scales, respectively (See in Figure A1).

**FIGURE A2:**
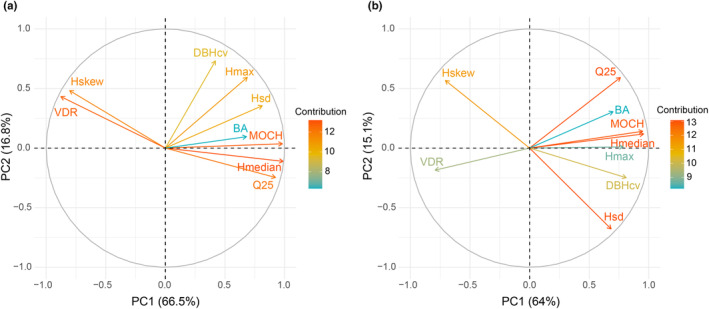
Results of PCA of nine plot‐level forest structural parameters at local (a) and regional (b) scales. Numbers in parentheses represent the percentages of the total variation accounted by the corresponding principal component. The abbreviations of variables followed Table A3.

A canopy height model (CHM) with 1 m resolution for each plot was derived by normalized point clouds using point‐to‐raster algorithms. Based on the normalized point clouds and CHM, we obtained a suite of plot‐level lidar‐derived metrics to characterize forest structure, including maximum canopy height, median canopy height, mean outer canopy height (MOCH), vertical distribution ratio (VDR), height standard deviation, height skewness, and 25% height quantile. In addition to lidar‐derived metrics, we calculated two widely used and ecologically important forest structure attributes based on plot inventory data: tree size variation within a plot as calculated by the coefficient of variation of individual DBH (DBHcv) and stand basal area (BA, the sum of stem basal area in each plot; Chu et al., 2019; Thom et al., 2021). In total, we obtained nine plot‐level forest structural parameters to summarize the forest structure in each plot. Descriptions of these parameters are presented in Table A3. CHM and the lidar‐derived metrics were implemented using the R package “lidR.” To simplify the following analyses and avoid collinearity, these metrics were integrated by PCA to obtain a comprehensive variable characterizing forest structure. The first axis explains over 60% of the total variation in these metrics at local and regional scales (See in Figure A2). Therefore, we used the two PC1s to represent forest structure at two scales in subsequent analyses.

Spatial structure plays an important role in plant community studies and is commonly used to predict diversity patterns (Dray et al., 2012). To account for spatially structured processes, from the centroid coordinates of each plot (i.e., *x* and *y*), we derived two sets of spatial structure variables at two scales. The first set was composed of the five terms of a trend surface polynomial (*x*, *y*, *x* ^*2*^, *y* ^*2*^, xy), which represent linear and curvilinear structures at plot extents across two scales. The second set was created employing the distance‐based Moran's eigenvector maps method (dbMEM). It decomposes Euclidean geographic distances between plots into a group of orthogonal spatial variables that denote the spatial relationship among plots (Borcard et al., 2004). The dbMEM spatial structure variables were acquired using the function *dbmem* in the “adespatial” package (Dray et al., 2022).

### 2.4 Beta‐diversity and turnover‐nestedness decomposition

We used Baselga's additive partitioning framework based on pairwise dissimilarity to decompose turnover and nestedness components from multidimensional total beta‐diversity (Baselga, 2010, 2013). We chose abundance‐based Sørensen dissimilarities (i.e., percentage difference dissimilarities) as index of total taxonomic beta‐diversity (β_sor_) and divided them into species turnover (β_sim_) and nestedness (β_sne_) components. In analogy with taxonomic dimension, total functional beta‐diversity (β_funsor_) based on calculations of convex hull volume in multidimensional functional space can be partitioned into functional turnover (β_funsim_) and functional nestedness (β_funsne_) components (Villéger et al., 2008, 2013), and total phylogenetic beta‐diversity (β_physor_) calculated using branch lengths of phylogenetic tree also allowing for turnover‐nestedness decomposition (denoted by β_physim_ and β_physne_; Leprieur et al., 2012). Before calculating beta‐diversity and turnover‐nestedness decomposition at two scales, we removed individuals with DBH greater than the 90% quantile of all DBH values (i.e., individuals in the canopy) to avoid using forest structure to explain their compositional variation. Pairwise dissimilarity partitioning approaches were performed using the “betapart” R package (Baselga et al., 2022).

### 2.5 Statistical analyses

Given that turnover and nestedness combined equal total beta‐diversity (i.e., dissimilarity), and similarity equals 1—dissimilarity, and according to Legendre (2014), we used a triangular plot to display the distribution of similarity, turnover, and nestedness of beta‐diversity for three dimensions at local and regional scales. Each triangle side represents one of the components. Triangular plots were created using the R package “ggtern.” The statistical comparisons of components between the two scales were examined using two‐sided Wilcoxon rank‐sum test. We used the relative proportions of turnover and nestedness in overall beta‐diversity to assess the relative contribution of the two components.

To determine the extent to which forest structure explains beta‐diversity and its components, we applied the modified version of distance‐based redundancy analysis (dbRDA; McArdle & Anderson, 2001). In essence, dbRDA as a multifactorial linear model, which enables testing the direct relations between a response matrix and a group of explanatory variables (Legendre & Anderson, 1999). It performs best when the response matrix has the Euclidean property, as the principal coordinates produced by a non‐Euclidean response matrix contain several negative eigenvalues and complex axes. When these complex ordination axes are ignored, the total sum‐of‐squares of the non‐Euclidean response is inflated, thus impacting the explanatory power of the model (Legendre, 2014; Moura et al., 2017). To avoid this problem, McArdle and Anderson (2001) presented a method to properly test the significance of the relation of a non‐Euclidean response with multiple explanatory variables and further proposed corrected statistics for dbRDA when a non‐Euclidean response is used. In particular, we used the adjusted *R* ^2^ derived from dbRDA to quantify the magnitude of forest structure impacts on beta‐diversity and its components in three dimensions at two scales.

To assess the relative importance of forest structure, topography, and spatial structure in explaining beta‐diversity and its components, dbRDA and associated variation partitioning analyses were applied (Peres‐Neto et al., 2006). In order to select forest structural parameters, topographic variables, and spatial structure that explain beta‐diversity and its components significantly, forward selection procedures were carried out. Forward selection considers two stopping rules (Blanchet et al., 2008): (a) the significance level α (<0.05) and (b) the adjusted *R* ^2^ exceeding the adjusted *R* ^2^ of the full model. After forward selection, variance partitioning was applied to evaluate the relative explanation of each predictor group, quantifying the unique and joint contributions (quantified by the adjusted *R* ^2^ derived from dbRDA) of forest structure, topography, and spatial structure to beta‐diversity and its component patterns. A modified version of dbRDA was accomplished with the *dbRDA.D* function from Legendre (2014) while others were completed with the “vegan” package (Oksanen et al., 2022). All analyses were performed using R version 4.2.0 (R Core Team, 2022).

## 3 RESULTS

**FIGURE 2:**
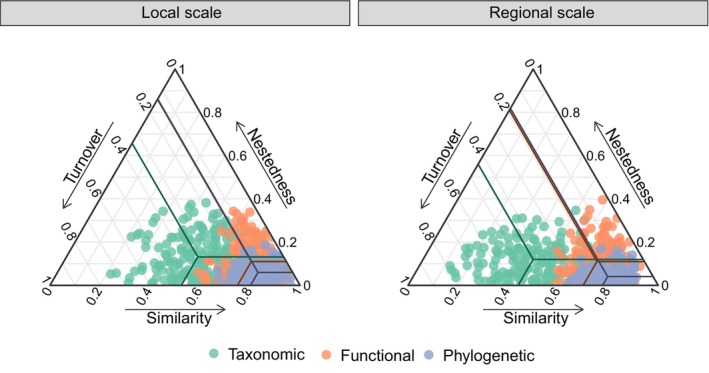
Triangular plots of the distribution of turnover, nestedness, and similarity for taxonomic, functional, and phylogenetic pairwise beta‐diversity at local and regional scales. Each point (with the same color) represents a pair of sites. Its position is determined by the values from the similarity (1—dissimilarity), turnover, and nestedness of the corresponding dimensions. For each dimension, the line in the plots is the centroid value. The separate triangular plots for each dimension of beta‐diversity are shown in Figure A3.

**FIGURE 3:**
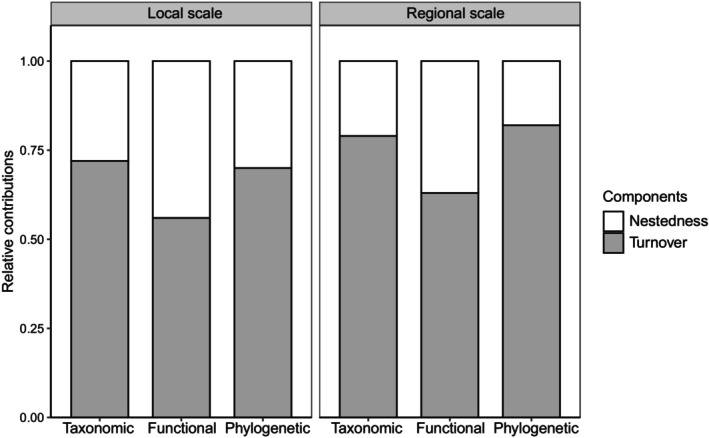
Relative contributions of turnover and nestedness components to total taxonomic, functional, and phylogenetic beta‐diversity at local and regional scales.

**FIGURE A3:**
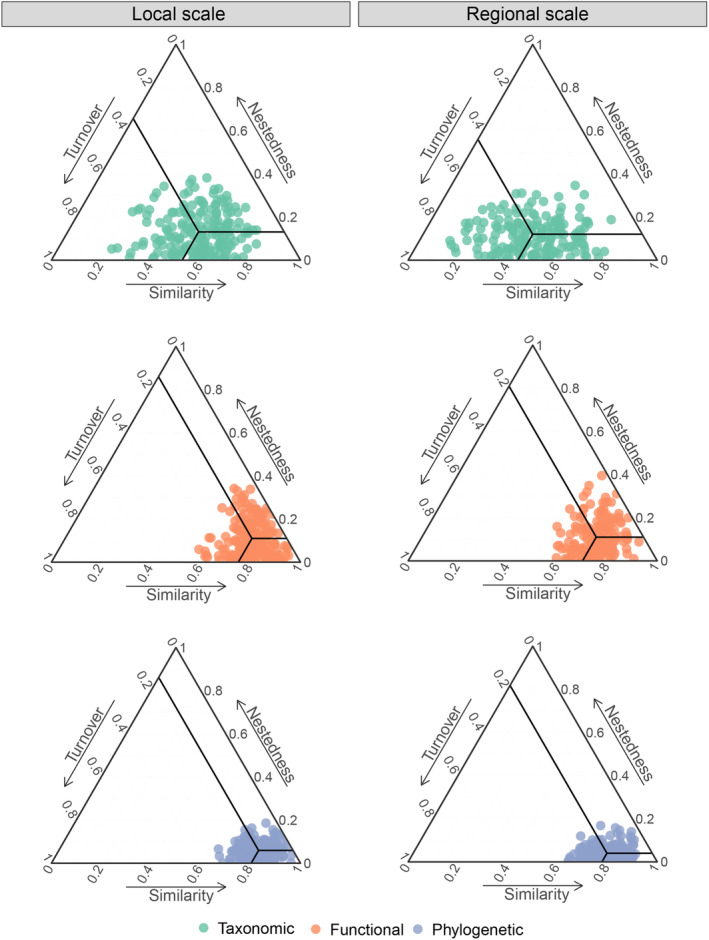
The separate triangular plots of the distribution of turnover, nestedness, and similarity for taxonomic, functional, and phylogenetic pairwise beta‐diversity at local and regional scales. Each point represents a pair of sites. Its position is determined by the values from the similarity (1– dissimilarity), turnover, and nestedness of the corresponding dimensions. For each dimension, the line in the plots is the centroid value.

According to the triangular plots, we found similar distributions of turnover, nestedness, and similarity for functional and phylogenetic dimensions at local and regional scales (i.e., low values of turnover and nestedness and high values of similarity; Figure 2). However, at the local scale, there were higher similarity and lower turnover for the taxonomic dimension than at the regional scale (Wilcoxon rank‐sum test: *n* = 361, *W* = 21,808, *p* < .001 and *n* = 361, *W* = 9743, *p* < .001 respectively; Figure 2; Figure A3). Additionally, the relative contributions of turnover and nestedness of total taxonomic, functional, and phylogenetic beta‐diversity exhibited consistent patterns at two scales, whereby turnover components were relatively higher than nestedness components (i.e., relative proportions > 0.50; Figure 3), indicating that turnover components dominated beta‐diversity for all three dimensions in subtropical forests. Notably, for nestedness components on functional dimension, their relative contributions to beta‐diversity were slightly lower than those of turnover at local and regional scales (0.44 and 0.37, respectively; Figure 3).

**FIGURE 4:**
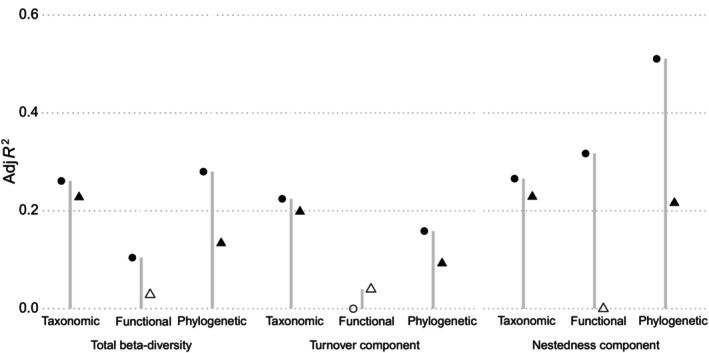
Variations in the taxonomic, functional, and phylogenetic beta‐diversity and its turnover and nestedness components explained by forest structure at local and regional scales, as quantified by adjusted *R* ^*2*^. Circles and triangles represent local and regional scales, respectively. Filled circles and triangles indicate significant effects (*p* < .05).

The dbRDA analyses show that explanations of forest structure for patterns of beta‐diversity and its components at the local scale were higher than those at the regional scale, especially for the phylogenetic dimension (Figure 4). Specifically, for the taxonomic dimension, the explanatory strength of forest structure for the patterns of beta‐diversity and its components was approximately 0.20 at both scales (Figure 4). For the functional dimension, however, forest structure only weakly explained all components except for nestedness at the local scale (Figure 4).

**FIGURE 5:**
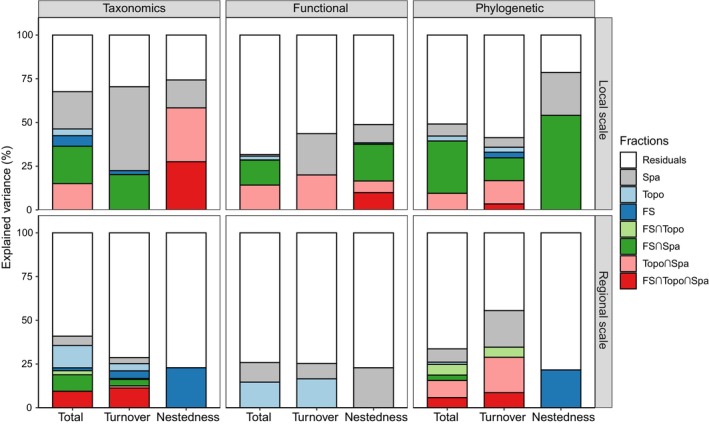
Variation partitioning results based on dbRDA showing variations in the taxonomic, functional, and phylogenetic beta‐diversity and its turnover and nestedness components explained by unique and joint effects of forest structure, topography, and spatial structure at local and regional scales. Variations explained by the different explanatory variables are shown. The explained variation is based on adjusted *R* ^*2*^ (%). Fractions with a “∩” sign indicate joint contributions of two or more variables. Abbreviations: FS, forest structure; Spa, spatial structure (dbMEM spatial variables); Topo, topography.

Variation partitioning based on dbRDA results indicated that the pure and joint fractions of forest structure, topography, and spatial structure were distinct in each dimension component at local and regional scales (Figure 5). Generally, the explained fraction of each component was higher at the local scale compared to the regional scale. At the local scale, pure spatial structure (Spa fraction) had some explanatory effect on the components, especially for the taxonomic dimension. Meanwhile, spatial forest structure (FS∩Spa fraction), as well as topographic spatial structure (Topo∩Spa fraction), explained several components, especially for taxonomic and phylogenetic dimensions (Figure 5). At the regional scale, by contrast, for the total beta‐diversity of taxonomic and functional dimensions, the proportion of pure topography (Topo fraction) was the highest among all the fractions; for the nestedness of taxonomic and phylogenetic dimensions, only pure forest structure (FS fraction) had an explanatory role, approximately 22% (Figure 5).

## 4 DISCUSSION

### 4.1 Turnover and nestedness in taxonomic, functional, and phylogenetic beta‐diversity

Our results suggest that turnover (i.e., changes in species identities and functional and phylogenetic attributes between sites) were in our subtropical forests generally more common than loss or gain of species and their functional and phylogenetic attributes (i.e., nestedness). This pattern was similar for the local and regional scale. Our analysis yielded similar results to numerous previous studies carried out on many different ecosystem types and taxa (Branco et al., 2020; Viana et al., 2016; Wang et al., 2018; Zhao et al., 2021). A meta‐analysis of turnover‐nestedness decomposition across taxa and ecosystems shows that turnover component is consistently the greater part of total beta‐diversity (Soininen et al., 2018). We provide further evidence for this conclusion from a multidimensional perspective. However, one notable result was about functional dimension at two scales, in which the proportions of nestedness to total beta‐diversity were higher than other dimensions, although still smaller than turnover (Figure 3). This means that functional nestedness in our study was also important in functional beta‐diversity. Other studies have also reported similar conclusions (e.g., Branco et al., 2020; Zhao et al., 2021).

We found higher similarity and lower turnover in taxonomic dimension at the local scale compared with the regional scale. This shows a scale‐dependent effect on the pattern of taxonomic total beta‐diversity, which is a generally accepted pattern (Condit et al., 2002; Nekola & McGill, 2014; Soininen et al., 2007; Figure 2). Probably due to spatial limitations in our study, we failed to detect scale‐dependent effects on patterns of functional and phylogenetic dimensions. A systematic assessment of the spatial scaling of beta‐diversity by Antão et al. (2019) noted that the turnover component, a major part of beta‐diversity, was closely in line with total beta‐diversity patterns, yet the nestedness component was mainly insensitive to scale changes. However, the multiple‐scale patterns of functional and phylogenetic beta‐diversity and their respective components remain still unclear, so the underlying scale effects need to be investigated further.

### 4.2 Drivers of taxonomic, functional, and phylogenetic beta‐diversity

In this study, we investigated the effects of topography, forest structure, and spatial structure on patterns of multidimensional beta‐diversity and its components in the same subtropical forest ecosystem but at varying scales. Environmental factors (i.e., topography and forest structure) tended to display stronger spatial autocorrelation at the local scale, which may be due to our 1‐ha study plots being continuous at the community level. At the local scale, pure spatial structure and its joint effects with topography and forest structure were the main factors driving all beta‐diversity facets and their respective components. Conversely, the contribution of spatial structure was relatively low at the regional scale (except for the functional dimension and β_physim_), while pure topography and pure forest structure became important explanatory variables. Our comparative analysis at distinct scales in the same ecosystem offers a broader view of the drivers of multidimensional beta‐diversity and its component patterns, as well as their scale effects. Moreover, beta‐diversity partitioning helps us to better understand the mechanisms that drive the observed beta‐diversity patterns, and as our results illustrate, different drivers may act on the different component, which in turn affects total beta‐diversity.

Many previous studies of plant community compositional variation have focused mainly on determining the relative contribution of environmental factors (i.e., environmental filtering) and pure spatial structure (i.e., dispersal limitation or neutral processes) in explaining beta‐diversity patterns (e.g., Baldeck et al., 2016; Legendre et al., 2009). Here, we found that with the exception of some components (β_funnes_ at the regional scale; β_sim_ and β_funsim_ at the local scale), environmental conditions (i.e., topography and forest structure) contribute more to beta‐diversity patterns than pure spatial variation. This implies that environmental filtering plays a critical role in determining the different facets of beta‐diversity of subtropical forests in Ailaoshan, which shows a rugged topography. Several studies have been reported on drivers of multifaceted beta‐diversity patterns in subtropical forest communities. For taxonomic dimension, our findings are consistent with Legendre et al. (2009) in subtropical forests and Baldeck et al. (2016) in tropical forests. Notably, for some components (β_funnes_ at the regional scale; β_sim_ and β_funsim_ at the local scale), the pure spatial structure had more explanatory power than environmental variables. This suggests that for these components, neutral processes play an important role in shaping patterns. Considering that our study was conducted on tree species of ≥10 cm DBH and that the distribution patterns of small‐diameter species are more subject to dispersal limitation (Asefa et al., 2019), the relative contribution of environmental filtering and neutral processes may turn out to be different for small‐diameter species. Furthermore, caution should be taken when inferring processes grounded on variation partitioning results, as the spatial structure of the environment may carry a dispersal process signal when dispersal spatially coincides with certain environmental variables (Chang et al., 2013), and pure spatial structure contains unobserved spatially structured environmental factors (e.g., soil properties; Gilbert & Bennett, 2010). Still, variation partitioning into environmental control and spatial components, as well as combining additive partitioning of multidimensional beta‐diversity, is considered an important tool for analyzing processes that lead to compositional variation of pairwise communities (Dray et al., 2012; López‐Delgado et al., 2020; Smith & Lundholm, 2010).

### 4.3 The roles of forest structure in shaping beta‐diversity

Forest structure regulates the configuration of nutrients and resources within the forest canopy, which consequently affects species distribution and diversity (Helbach et al., 2022; Tang & Dubayah, 2017). Numerous researches have investigated how forest structure drives multidimensional alpha‐diversity patterns. However, its role in beta‐diversity and its two complementary components remains to be investigated (Gough et al., 2020; Zhang et al., 2022). Adopting multidimensional and multiple‐scale perspectives, we used integrated forest structure to explain beta‐diversity and its component patterns and then explored the drivers of these patterns. We found that forest structure plays a significant role in shaping multidimensional beta‐diversity patterns. Moreover, the PCA results reveal that forest structure is a combination of light heterogeneity (characterized by Hsd and Hskew) and light availability (characterized by MOCH and VDR), reflecting canopy light environment of plots (Figure A2; Table A4). At the local scale, forest structure influences beta‐diversity and its component patterns (except for β_funsim_) mainly through its spatial structure fraction. Interestingly, at the regional scale, forest structure drives nestedness patterns of taxonomic and phylogenetic dimensions to some extent. On the one hand, the higher turnover among the plots may result from rare species inhabiting different plots, whose distribution is less affected by forest structure. On the other hand, the dominant canopy trees in the Ailaoshan subtropical forests primarily belong to three families (Fagaceae, Theaceae, and Lauraceae), resulting in a relatively consistent abundance and lineage composition of dominant species across plots. Therefore, the relatively low degree of taxonomic and phylogenetic nestedness can be attributed to the forest structure. This suggests that light environment variation between sites influences the gain or loss of community composition and phylogenetic lineages between sites, which in turn shapes nestedness patterns, providing new insights into the study of nestedness patterns. Additionally, our results suggest that by considering forest structure as a light environmental variable, the effect of niche processes would almost certainly be better captured across dimensions and components. This is in accordance with other works evaluating the significance of the relevant variables of forest structure on diversity patterns (e.g., Hubbell et al., 1999; Zhang et al., 2016, 2022).

Although our study detected a unique effect on forest structure in driving beta‐diversity and its component patterns, there are still many challenges for further study. For example, various aspects of forest structure (vertical, horizontal, external, internal) likely have different functions in driving diversity patterns (Gough et al., 2020). In future studies, it is possible to develop indicators to distinguish these roles to better understand mechanisms. Moreover, forest structure measures need to be directly linked to ecological processes, such as asymmetric light competition and disturbance dynamics (Cushman et al., 2022; Yi et al., 2022). It has been shown that the canopy structure (e.g., closure and gap fraction) estimated from airborne LiDAR data is a practical indicator of light resource availability (Alexander et al., 2013). The rapid development of near‐ground remote sensing and fine‐scale drone technology has enabled the gathering of high‐quality information on canopy and internal structure, providing a clearer view of the vertical, horizontal, and internal niche differentiation of the forest, greatly facilitating biodiversity assessment and disturbance dynamics detection (Anderson & Gaston, 2013). Furthermore, UAV LiDAR technology coupled with long‐term monitoring of forest dynamic plots will provide a more holistic understanding of multidimensional diversity maintenance and dynamics.

## AUTHOR CONTRIBUTIONS

**Zhiliang Yao:** Conceptualization (equal); data curation (equal); formal analysis (lead); investigation (equal); methodology (lead); writing – original draft (lead); writing – review and editing (equal). **Xin Yang:** Conceptualization (equal); data curation (equal); investigation (equal); methodology (lead); writing – original draft (equal). **Bin Wang:** Data curation (equal); investigation (equal). **Xiaona Shao:** Data curation (equal); investigation (equal). **Handong Wen:** Data curation (equal); investigation (equal). **Yun Deng:** Data curation (equal); investigation (equal); resources (equal). **ZhiMing Zhang:** Data curation (equal); investigation (equal); resources (equal). **Min Cao:** Data curation (equal); investigation (equal); resources (equal). **Luxiang Lin:** Conceptualization (lead); data curation (equal); funding acquisition (lead); resources (equal); writing – review and editing (lead).

## FUNDING INFORMATION

This study was supported by the Strategic Priority Research Program of the Chinese Academy of Sciences, Grant No. XDB31000000, the Joint Fund of the National Natural Science Foundation of China‐Yunnan Province (U1902203), and Southeast Asia Biodiversity Research Institute, Chinese Academy of Sciences (151C53KYSB20200019).

## CONFLICT OF INTEREST STATEMENT

The authors have no competing interests to declare.

## Data Availability

The data that support the findings of this study are available from the Dryad Digital Repository. DOI: https://doi.org/10.5061/dryad.4qrfj6qgf

## APPENDIX A

**TABLE A1:** Loadings of 11 functional traits to the first four PCoA axes, and cumulative proportion (in percentage) of the four PCoA axes at local and regional scales.

Traits	Local scale				Regional scale			
	PCoA1	PCoA2	PCoA3	PCoA4	PCoA1	PCoA2	PCoA3	PCoA4
leaf C	0.02	0.65	0.20	0.63	−0.07	−0.25	0.70	0.56
leaf N	0.78	0.13	−0.23	0.18	0.80	−0.14	0.05	0.28
leaf P	0.91	−0.10	−0.11	0.20	0.91	0.06	−0.02	0.17
leaf K	0.65	−0.33	0.12	0.43	0.71	0.27	0.06	0.24
WD	−0.23	−0.10	−0.71	0.15	−0.18	−0.35	−0.54	0.37
LA	0.59	0.17	0.49	−0.19	0.22	0.33	0.65	−0.33
LCC	−0.34	−0.49	0.16	0.59	−0.09	0.72	0.07	0.34
LT	−0.60	−0.27	0.57	0.10	−0.52	0.71	0.10	0.19
SLA	0.90	−0.15	−0.17	−0.14	0.89	−0.17	−0.17	−0.19
LDMC	−0.40	0.68	−0.42	0.15	−0.44	−0.73	0.22	0.15
*MaxH*	0.26	0.57	0.47	−0.06	0.07	−0.30	0.74	−0.14
Variance	3.77	1.73	1.63	1.10	3.34	2.06	1.85	0.96
Cumulative proportion	34.25	50.00	64.80	74.82	30.38	49.11	65.92	74.69

Abbreviations: LA, leaf area; LCC, leaf chlorophyll content; LDMC, leaf dry matter content; leaf C, leaf carbon content; leaf K, leaf potassium content; leaf N, leaf nitrogen content; leaf P, leaf phosphorus content; LT, leaf thickness; *MaxH*, tree maximum height; SLA, specific leaf area; WD, wood density.

**TABLE A2:** Descriptions of topographic variables in this study.

Parameters	Abbreviations	Descriptions
Mean elevation	meanelev	Mean elevation of 100 m^2^ quadrats within the plot
Topographic position index	TPI	Mean TPI of 100 m^2^ quadrats within the plot
Topographic wetness index	TWI	Mean TWI of 100 m^2^ quadrats within the plot
Solar radiation aspect index	Solar_rad	Mean solar radiation of 100 m^2^ quadrats within the plot
Topographic complexity	complexity	The ratio of the surface area to the projected area of the plot

**TABLE A3:** Descriptions of forest structural parameters in this study.

Parameters	Abbreviations	Descriptions
Mean outer canopy height	MOCH	Mean of maximum height in 1 m^2^ grid of plot
Maximum canopy height	*H*_max_	Maximum height within the plot
Median canopy height	*H*_median_	Median height within the plot
Height standard deviation	Hsd	Standard deviation of heights within plot
Vertical distribution ratio	VDR	VDR = (*H*_max_−*H*_median_)/*H*_max_
Height skewness	Hskew	Skewness of heights within plot
25% height quantile	Q25	25% quantile of heights within plot
Stand basal area	BA	The sum of stem basal area within plot
Coefficient of variation of DBH	DBHcv	The coefficient of variation of tree DBH within plot

**TABLE A4:** Contributions (in percentage) of nine plot‐level forest structural parameters to the first principal component (PC1) at local and regional scales. The abbreviations of variables followed Table A3.

Parameters	Local scale PC1	Regional scale PC1
MOCH	16.12	15.65
*H*_max_	7.90	10.72
*H*_median_	16.33	15.91
Hsd	11.04	8.13
Q25	14.27	10.08
Hskew	10.79	8.66
VDR	12.85	10.92
BA	7.76	8.52
DBHcv	2.94	11.41
